# Addressing therapeutic options for KPC-3-producing ST307-*Klebsiella pneumoniae*: insights from *in vitro* evolution and mutant prevention strategies

**DOI:** 10.1128/aac.00488-26

**Published:** 2026-07-01

**Authors:** Marta Hernández-García, Blanca Pérez-Viso, Marta Nieto-Torres, María García-Castillo, Malkoa Michelena, Luis Martínez-Martínez, Fernando Baquero, Patricia Ruiz-Garbajosa, Rafael Cantón

**Affiliations:** 1 Servicio de Microbiología, Hospital Ramón y Cajal and Instituto Ramón y Cajal de Investigación Sanitaria (IRYCIS) 537482 , Madrid, Spain; 2 CIBER de Enfermedades Infecciosas (CIBERINFEC), Instituto Salud Carlos III (ISCIII) 38176, Madrid, Spain; 3 Unidad de Microbiología, Hospital Universitario Reina Sofia 16501, Córdoba, Spain; 4 Departamento de Química Agrícola, Edafología y Microbiología, Universidad de Córdoba, Córdoba, Spain; 5 Instituto Maimónides de Investigación Biomédica de Córdoba, Córdoba, Spain; University of Fribourg, Fribourg, Switzerland

**Keywords:** KPC-ST307-*K. pneumoniae*, ceftazidime-avibactam, meropenem-vaborbactam, imipenem-relebactam, collateral sensitivity to carbapenems

## Abstract

Ceftazidime-avibactam, meropenem-vaborbactam, and imipenem-relebactam are key therapeutic options for infections caused by KPC-producing *Klebsiella pneumoniae* (KPC-Kp). Resistance may emerge during therapy, but knowledge on mutant prevention and collateral effects remains limited. Killing kinetics assays of ceftazidime-avibactam, meropenem-vaborbactam, imipenem-relebactam, and the combination of ceftazidime-avibactam with meropenem (CZA + MER) were performed against clinical KPC-3-ST307-Kp isolates and ceftazidime-avibactam-resistant KPC-ST307-Kp mutants (KPC-31/KPC-46/KPC-53/KPC-61/KPC-62/KPC-66/KPC-92/KPC-150-Kp) with different genetic backgrounds. Single-step mutant frequencies and resistance trajectories were established. WGS was used to identify resistance mechanisms, and collateral resistance to other last-line agents was assessed. In time-kill assays, meropenem and ceftazidime-avibactam showed bactericidal activity at high concentrations (2× MIC and 4× MIC). Meropenem-vaborbactam was bactericidal only at high exposures (2× MIC and 4× MIC) and with reduced activity in porin-deficient strains, whereas imipenem-relebactam was bactericidal in half of the strains at 4× MIC. CZA + MER showed synergistic bactericidal activity across all KPC-Kp isolates, even at subinhibitory concentrations. CZA + MER completely suppressed the emergence of resistant mutants (mutation rate <10⁻¹⁰), while stepwise selection with ceftazidime-avibactam, meropenem-vaborbactam, and imipenem-relebactam rapidly led to resistance. Meropenem-vaborbactam and imipenem-relebactam selected mutants at lower concentrations and higher frequencies than ceftazidime-avibactam. Ceftazidime-avibactam resistance was mainly driven by *bla*_KPC_ diversification, whereas meropenem-vaborbactam and imipenem-relebactam selected truncating mutations in OmpK36. Imipenem-relebactam exerted stronger collateral effects than meropenem-vaborbactam, extending cross-resistance to aztreonam-avibactam and cefepime-taniborbactam, while cefiderocol activity remained largely preserved. Plasmid copy number contributed minimally to resistance phenotypes. Ceftazidime-avibactam, meropenem-vaborbactam, and imipenem-relebactam impose distinct evolutionary pressures on KPC-ST307-Kp, resulting in divergent resistance pathways and collateral resistance profiles. CZA + MER may offer a more sustainable strategy by preventing resistance emergence and limiting cross-resistance to last-line agents.

## INTRODUCTION

*Klebsiella pneumoniae*-producing KPC (KPC-Kp) represents one of the most critical challenges in current clinical microbiology and hospital infection control. KPC-Kp strains, often belonging to high-risk epidemic lineages, are responsible for numerous outbreaks associated with high morbidity, mortality, and with therapeutic options that, although expanding, remain limited in many settings because of geographic variability in drug availability and access (1, 2). Current guidelines recommend newly approved β-lactam/β-lactamase inhibitor (BL/BLI) combinations, such as ceftazidime-avibactam (CZA), meropenem-vaborbactam (MEV), and imipenem-relebactam (IMR), as preferred options for KPC-Kp infections (3–5). Overall, these combinations have been associated with high clinical success and favorable safety profiles compared with traditional regimens for carbapenem-resistant *Enterobacterales* (CRE) infections, including those caused by KPC-Kp (6–10).

Resistance to these novel BL/BLI combinations emerged soon after their introduction. Ceftazidime-avibactam resistance has been increasingly reported during or after therapy, most commonly due to KPC-2/KPC-3 variants with amino-acid substitutions that reduce avibactam binding (11, 12). These KPC-Kp mutants often display “collateral sensitivity” to carbapenems, suggesting a potential role for carbapenem monotherapy. However, the *in vivo* efficacy of this strategy remains uncertain, and combination therapy with other active agents is frequently recommended (13–16). In addition, ceftazidime-avibactam exposure may co-select *K. pneumoniae*-producing novel KPC variants that accumulate porin alterations, leading to cross-resistance to last-line agents targeted for infections due to MBL producers, including cefiderocol (FDC) and not-yet-approved BL/BLI combinations such as cefepime-taniborbactam (FTB) and cefepime-zidebactam (17–20). By contrast, meropenem-vaborbactam and imipenem-relebactam have shown good *in vitro* and clinical activity against KPC-Kp, including certain ceftazidime-avibactam-resistant isolates. However, resistance has also been described, typically involving increased *bla*_KPC_ copy number together with OmpK35/OmpK36 porin alterations (21–25).

Since 2017, the *K. pneumoniae* ST307 clone has predominated in our hospital, mainly associated with KPC production and, in many cases, with KPC variants conferring ceftazidime-avibactam resistance; most often in patients with prior ceftazidime-avibactam exposure (12, 18, 26). Some ST307 strains combining KPC variants and porin defects have also shown collateral resistance or increased MICs to meropenem-vaborbactam, imipenem-relebactam, cefepime-taniborbactam, aztreonam-avibactam (AZA), and cefiderocol (18, 26, 27). These findings underscore that the optimal therapy for KPC-Kp infections remains uncertain. Understanding resistance dynamics in the context of *in vitro* susceptibility and clinical antibiotic exposure is crucial to designing further effective treatment strategies.

In this work, we aimed to evaluate potential strategies to optimize therapy against KPC-ST307-Kp infections and to prevent the emergence and selection of resistant mutants, focusing on the novel BL/BLI combinations currently approved and recommended by clinical guidelines (ceftazidime-avibactam, meropenem-vaborbactam, and imipenem-relebactam). We also assessed the potential benefits of combining ceftazidime-avibactam and meropenem (CZA + MER) and the feasibility of carbapenem monotherapy in KPC-Kp mutants exhibiting collateral sensitivity. Finally, we investigated the emergence of collateral resistance to other last-line agents in KPC-ST307-Kp following exposure to ceftazidime-avibactam, meropenem-vaborbactam, imipenem-relebactam, and CZA + MER.

## RESULTS

### Antimicrobial susceptibility testing

A total of eight KPC-ST307-*K.pneumoniae* (KPC-ST307-Kp) clinical strains producing different KPC-3 variants were studied: KPC-31-ST307-Kp, KPC-46-ST307-Kp, KPC-53-ST307-Kp, KPC-61-ST307-Kp, KPC-62-ST307-Kp, KPC-66-ST307-Kp, KPC-92-ST307-Kp, and KPC-150-ST307-Kp. Three ST307-Kp isolates producing KPC-3 were also included. The resistance phenotypes and genotypes of all strains are indicated in Table 1 and Table S1, respectively. The baseline KPC-3-ST307-Kp isolates showed elevated/resistant MICs to carbapenems and retained susceptibility to novel agents. All strains carrying mutated KPC variants exhibited resistance to ceftazidime-avibactam (range: 32/4–128/4 mg/L). KPC-150-ST307-Kp combined truncated OmpK35 and OmpK36 porins; KPC-62-ST307-Kp had mutations in OmpA and EnvZ/OmpR porin proteins, and KPC-31-ST307-Kp contained an altered PBP2. These isolates displayed resistance or elevated MICs to carbapenems, meropenem-vaborbactam, imipenem-relebactam, cefiderocol, cefepime-taniborbactam, and aztreonam-avibactam. The remaining KPC-Kp mutants carried porin proteins and PBPs identical to those of the reference ST307-*K. pneumoniae* NCTN00000000 and exhibited collateral sensitivity to carbapenems and remained susceptible to other novel antimicrobials (Table 1). The antimicrobial susceptibility results of *bla*_KPC_ variants cloned into the isogenic *E. coli*-TOP10 strain are shown in Table S2.

**TABLE 1:** Antimicrobial susceptibility results obtained from KPC-ST307-Kp clinical isolates selected for the study^*a*^

Isolate	MIC values (mg/L)													KPC mutation
	PT4	FOT	TAZ	CZA	MER	MEV	IMI	IMR	AZT	AZA	FEP	FTB	FDC	
KPC-31-ST307-Kp	>32/4	>32	>512	128/4	0.12	≤0.06/8	≤0.25	0.25/4	>32	2/4	32	0.5/4	4	D179Y
KPC-46-ST307-Kp	>32/4	>32	512	64/4	≤0.06	≤0.06/8	≤0.25	0.25/4	>32	0.5/4	16	1/4	1	L168P
KPC-53-ST307-Kp	>32/4	>32	256	64/4	0.12	≤0.06/8	≤0.25	0.25/4	>32	≤0.25/4	32	0.5/4	1	L167E168dup
KPC-61-ST307-Kp	>32/4	>32	256	64/4	0.25	≤0.06/8	≤0.25	0.12/4	>32	≤0.25/4	32	0.25/4	1	S170P
KPC-62-ST307-Kp	>32/4	>32	>512	32/4	8	1/8	8	2/4	>32	1/4	>32	8/4	8	L168Q
KPC-66-ST307-Kp	32/4	>32	256	64/4	≤0.06	≤0.06/8	≤0.25	0.25/4	>32	≤0.25/4	16	0.5/4	1	E167_L168del
KPC-92-ST307-Kp	32/4	>32	256	64/4	0.12	≤0.06/8	≤0.25	0.25/4	>32	≤0.25/4	16	0.5/4	1	E167_N169delinsD
KPC-150-ST307-Kp	>32/4	>32	>512	64/4	8	4/8	2	2/4	>32	2/4	>32	16/4	4	L166_N169delinsH
KPC-3-ST307-Kp (lineage 1)	>32/4	>32	256	2/4	8	≤0.06/8	4	0.25/4	>32	≤0.25/4	>32	0.25/4	0.5	–^*b*^
KPC-3-ST307-Kp (lineage 2)	>32/4	>32	256	4/4	16	≤0.06/8	8	0.25/4	>32	≤0.25/4	>32	0.5/4	1	–
KPC-3-ST307-Kp (lineage 3)	>32/4	>32	128	2/4	8	≤0.06/8	8	0.25/4	>32	≤0.25/4	>32	0.5/4	0.25	–

^*a*^ PT4, piperacillin-tazobactam; FOT, cefotaxime; TAZ, ceftazidime; CZA, ceftazidime-avibactam; MER, meropenem; MEV, meropenem-vaborbactam; IMI, imipenem; IMR, imipenem-relebactam; AZT, aztreonam; AZA, aztreonam-avibactam; FEP, cefepime; FTB, cefepime-taniborbactam; FDC, cefiderocol.

^*b*^ –, mutations not found.

### Killing kinetics studies

**Fig 1:**
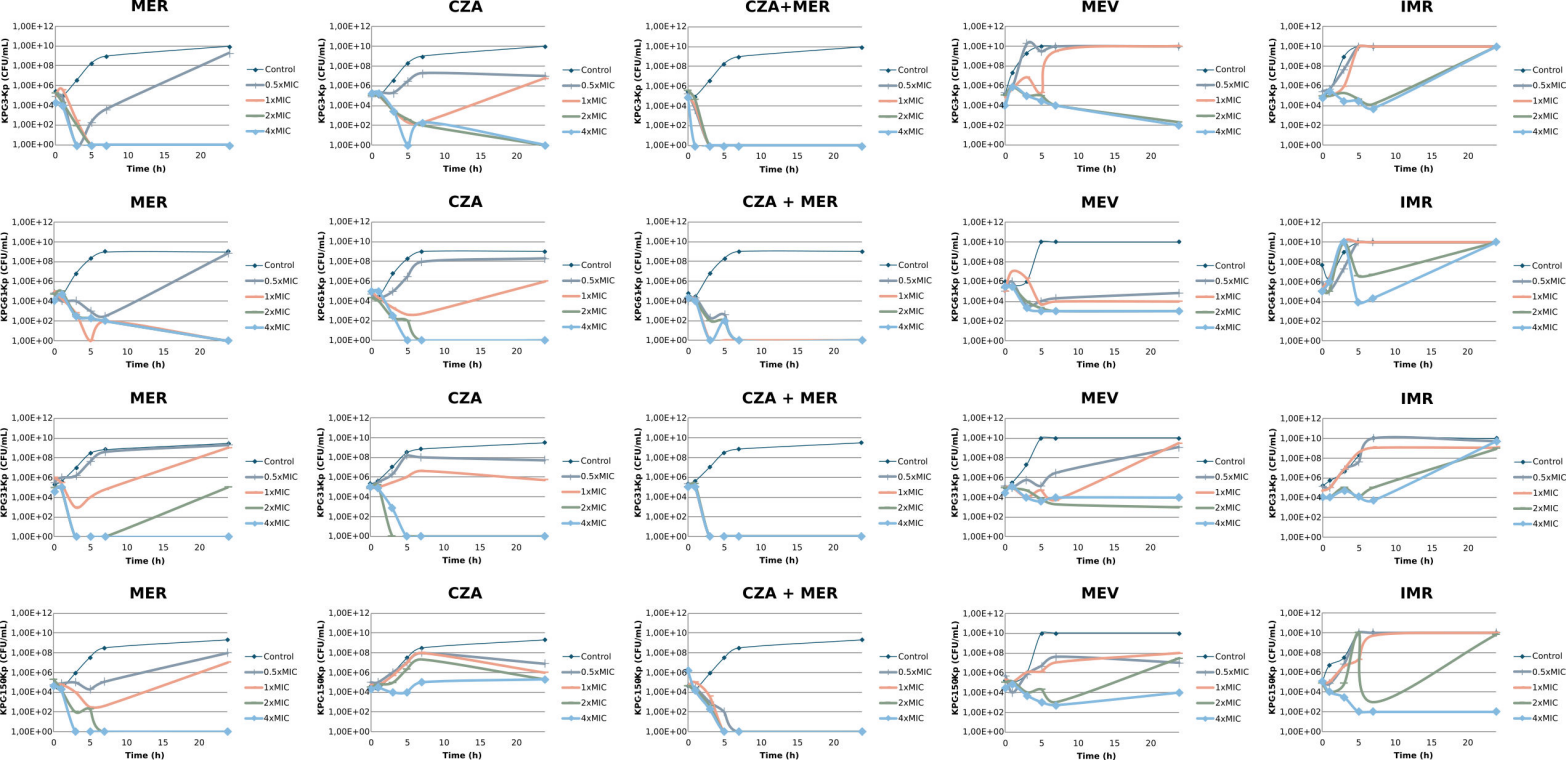
Results of time-kill assays of KPC-3-ST307-Kp, KPC-61-ST307-Kp, KPC-31-ST307-Kp (PBP2-A523T), and KPC-150-ST307-Kp (truncated OmpK36 and OmpK35 porins) strains exposed to MER, CZA, ceftazidime-avibactam in combination with meropenem (CZA + MER; ratio 1:1), MEV, and IMR at increasing concentrations (0.5× MIC, 1× MIC, 2× MIC, and 4× MIC). Growth control represents the mean value (log_10_ CFU/mL) of all strains in the absence of antibiotics. For all assays, the initial inoculum was ∼10^5^ CFU/mL.

To evaluate the activity of ceftazidime-avibactam, meropenem, CZA + MER (ratio 1:1), meropenem-vaborbactam, and imipenem-relebactam, time-kill assays were performed with all KPC-ST307-Kp mutants and one baseline KPC-3-ST307-Kp isolate (Fig. 1; Fig. S1).

Meropenem exhibited bactericidal activity at 1× MIC in five of nine strains within 3 h and at 2× MIC in the remaining four strains within 3–5 h. Ceftazidime-avibactam showed bactericidal activity within 3 h at subinhibitory concentrations (0.5× MIC) in four strains, at 1× MIC in two strains, at 2× MIC in two strains, and within 5 h at 4× MIC in one strain (KPC-150-Kp, which harbors truncated porins). Interestingly, the combination of CZA + MER (1:1 ratio) demonstrated bactericidal activity at subinhibitory concentrations in all strains, within 3 h in eight isolates and within 5 h in the remaining isolate (KPC-150-Kp). Imipenem-relebactam was bactericidal in four isolates (KPC-53-Kp, KPC-62-Kp, KPC-66-Kp, and KPC-150-Kp) at 1× MIC or 2× MIC within 3–5 h, and at 4× MIC in KPC-150-Kp within 3 h. Meropenem-vaborbactam achieved bactericidal activity at subinhibitory concentrations in six of nine strains within 5–7 h, at 1× MIC or 2× MIC within 5 h in two strains, and at 2× MIC in KPC-150-Kp within 7 h (Fig. 1; Fig. S1).

**Fig 2:**
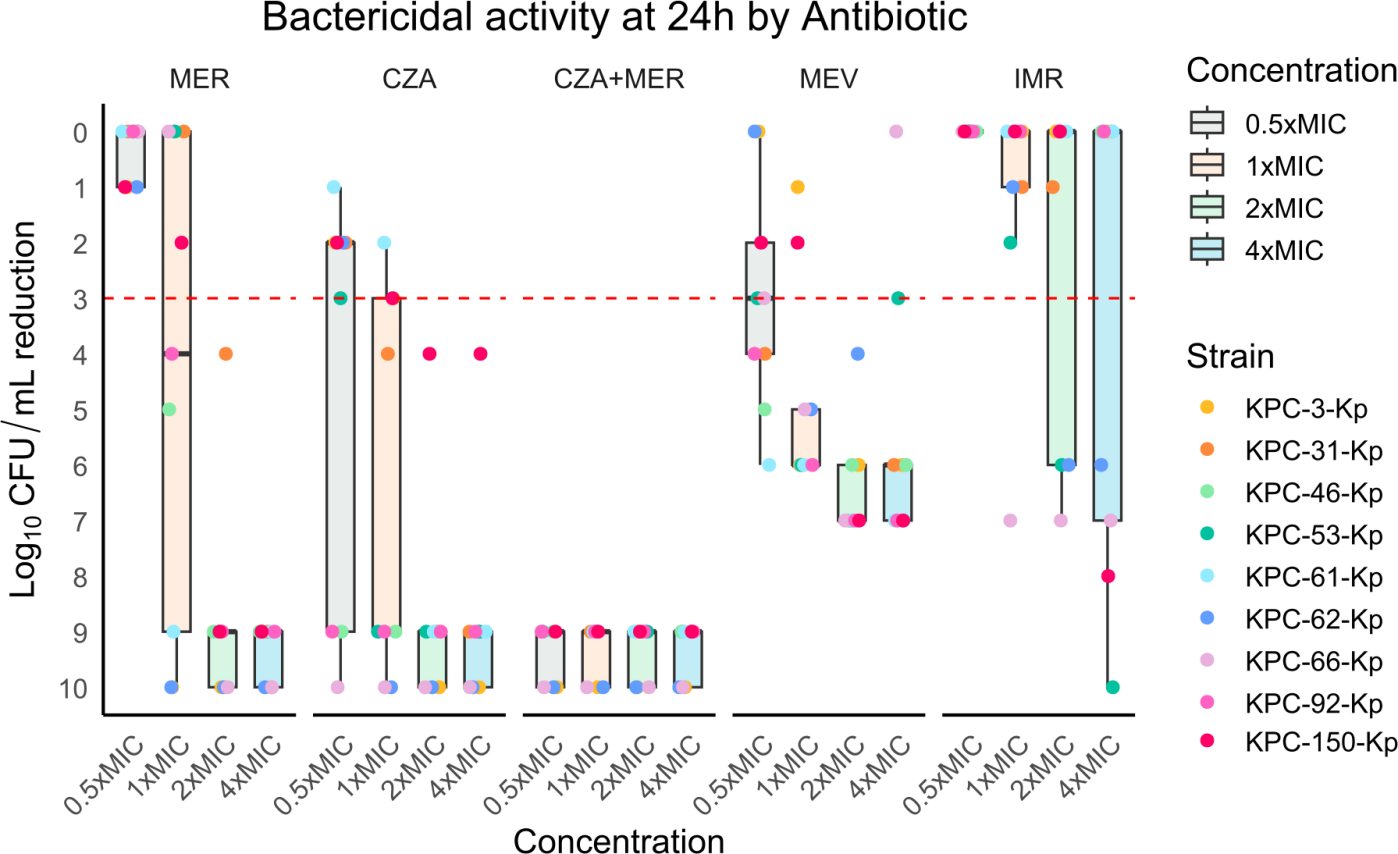
Distribution of log_10_ reductions in CFU/mL at 24 h for each antibiotic (meropenem, MER; ceftazidime-avibactam, CZA; ceftazidime-avibactam in combination with meropenem, CZA + MER [ratio 1:1]; meropenem-vaborbactam, MEV; and imipenem-relebactam, IMR) and concentration (0.5× MIC, 1× MIC, 2× MIC, and 4× MIC). The boxes represent the interquartile range (IQR), spanning from the first quartile (Q1) to the third quartile (Q3), with the horizontal line inside the box indicating the median (Q2). The whiskers extend to the most extreme values within 1.5 × IQR. Each colored point represents the individual value of each KPC-ST307-*K. pneumoniae* strain. The dashed red line indicates the threshold for bactericidal activity (≥3 log_10_ reduction).

Figure 2 shows the bactericidal activity (log_10_ reduction of CFU/mL) at 24 h of each of the antibiotics at the different concentrations tested (0.5× MIC, 1× MIC, 2× MIC, and 4× MIC). Overall, meropenem was bactericidal at 1× MIC in several strains and consistently exceeded the ≥3 log_10_ reduction threshold at 2× MIC and 4× MIC in all isolates. Ceftazidime-avibactam was more variable, and bactericidal activity was only observed at the highest concentrations (2× MIC and 4× MIC). The combination of CZA + MER displayed potent activity even at 0.5× MIC (≥3 log_10_ reduction in all isolates), highlighting strong synergy between these antibiotics. Meropenem-vaborbactam was bactericidal primarily at higher concentrations (2× MIC and 4× MIC), while at 0.5× MIC, almost all strains were below or just at the threshold. Imipenem-relebactam exhibited lower efficacy, achieving bactericidal activity in only about half of the isolates at 4× MIC. In those strains where no bactericidal effect of meropenem-vaborbactam and imipenem-relebactam was observed at 24 h, regrowth was detected after 7 h at high concentrations (2× MIC and/or 4× MIC) (Fig. 1; Fig. S1).

### Synergy testing

The synergy of ceftazidime-avibactam in combination with MER (ratio 1:1) was determined in all KPC-ST307-Kp mutants and one KPC-3-ST307-Kp parental strain by the MIC strip synergy test. Table 2 shows the MICs of CZA + MER for each strain and the resulting FIC index. All KPC-Kp mutants were susceptible to the combination CZA + MER with MICs ranging from 0.016/4 to 12/4 mg/L and FIC indices between 0.13 and 0.36, indicating synergistic activity.

**TABLE 2:** Results (FICI) of the MIC gradient synergy test for CZA and MER combination against KPC-3-ST307-Kp and KPC-ST307-Kp mutants

Isolate	MIC by Etest		Etest synergy test^*a*^		
	CZA (mg/L)	MER (mg/L)	CZA^*b*^ (mg/L)	MER^*b*^ (mg/L)	ΣFIC^*c*^
KPC-3-ST307-Kp (lineage 1)	1.5/4	1.5	0.016/4	0.19	0.14 (S)
KPC-31-ST307-Kp	256/4	0.19	12/4	0.032	0.22 (S)
KPC-46-ST307-Kp	6/4	0.05	0.75/4	0.004	0.21 (S)
KPC-53-ST307-Kp	8/4	0.06	2/4	0.023	0.45 (S)
KPC-61-ST307-Kp	32/4	0.19	6/4	0.032	0.36 (S)
KPC-62-ST307-Kp	32/4	6	2/4	0.38	0.13 (S)
KPC-66-ST307-Kp	8/4	0.12	2/4	0.012	0.35 (S)
KPC-92-ST307-Kp	12/4	0.06	2/4	0.016	0.43 (S)
KPC-150-ST307-Kp	64/4	12	2/4	1.5	0.16 (S)

^*a*^ MIC determined by MIC gradient strips crossed perpendicularly at the intersection between the MIC obtained by microdilution for each antibiotic.

^*b*^ MIC values of ceftazidime-avibactam and meropenem obtained in combination.

^*c*^ ΣFIC ≤ 0.5 was considered synergistic (S), 0.5 < ΣFIC ≤ 4 was considered indifferent (I), and ΣFIC > 4 was considered antagonistic (A).

### Single-step mutant selection and mutant prevention concentration assays

The frequency of mutant emergence was investigated in two parental KPC-3-ST307-Kp strains (lineages 2 and 3) exposed to increasing concentrations of ceftazidime-avibactam, CZA + MER (ratio 1:1), meropenem-vaborbactam, and imipenem-relebactam (Table 3). The mutant prevention concentration (MPC) of ceftazidime-avibactam was 8/4 mg/L in both isolates and showed the lowest mutation frequencies, 1.5 × 10⁻⁸ at 1× MIC and 7.8 × 10⁻⁹ at 2× MIC for lineages 2 and 3, respectively, with no detectable mutants at higher concentrations. The MPC of imipenem-relebactam was 4/4 mg/L in both parental isolates; low-medium mutation frequencies were detected at 8× MIC (3.6 × 10⁻⁷ and 3.3 × 10⁻⁷ for lineages 2 and 3, respectively), while no mutants emerged at 16× MIC. Regarding meropenem-vaborbactam, the MPC was the lowest (0.5/8 mg/L), but mutants appeared at 4× MIC in both isolates, with high mutation frequencies of 1.8 × 10⁻⁵ (lineage 2) and 1.8 × 10⁻⁶ (lineage 3). Remarkably, the CZA + MER combination (ratio 1:1) completely suppressed the emergence of resistant mutants at all tested concentrations (mutation rate <10^−10^).

**TABLE 3:** Frequency of emergence of mutants and MPCs of the baseline KPC-3-ST307-Kp clinical isolates exposed to incremental concentrations of CZA, MEV, and IMR^*a*^

Strain	CZA MIC (mg/L)	CZA concentration (mg/L)	CZA mutant frequency	MEV MIC (mg/L)	MEV concentration (mg/L)^*b*^	MEV mutant frequency	IMR MIC (mg/L)	IMR concentration (mg/L)^*b*^	IMR mutant frequency
KPC-3-ST307-Kp (lineage 2)	4/4	×1 (4/4)	≤1.5 × 10^−8^	≤0.06/8	×1 (0.06/8)	–	0.25/4	×1 (0.25/4)	–
		**×2 (8/4**)	0		×2 (0.12/8)	–		×2 (0.5/4)	–
		×4 (16/4)	0		×4 (0.25/8)	≤1.8 × 10^−5^		×4 (1/4)	–
		×8 (32/4)	0		**×8 (0.5/8**)	0		×8 (2/4)	≤3.6 × 10^−7^
		×16 (64/4)	0		×16 (1/7)	0		**×16 (4/4**)	0
KPC-3-ST307-Kp (lineage 3)	2/4	×1 (2/4)	–	≤0.06/8	×1 (0.06/8)	–	0.25/4	×1 (0.25/4)	–
		×2 (4/4)	≤7.8 × 10^−9^		×2 (0.12/8)	–		×2 (0.5/4)	–
		**×4 (8/4**)	0		×4 (0.25/8)	≤1.8×10^−6^		×4 (1/4)	–
		×8 (16/4)	0		**×8 (0.5/8**)	0		×8 (2/4)	≤3.3 × 10^−7^
		×16 (32/4)	0		×16 (1/8)	0		**×16 (4/4**)	0

^*a*^ –, countless colony growth. (0) No bacterial growth or mutants detected. Bold letters indicate the MPCs obtained for each antibiotic.

^*b*^ For ceftazidime-avibactam plus meropenem (1:1 ratio), concentrations refer to the MIC of ceftazidime in the combination, with avibactam fixed at 4 mg/L and meropenem adjusted to the same multiple of its MIC. Initial inoculum adjusted to 10⁶ CFU/mL for meropenem-vaborbactam and imipenem-relebactam.

### Dynamics of *in vitro* resistance development

**Fig 3:**
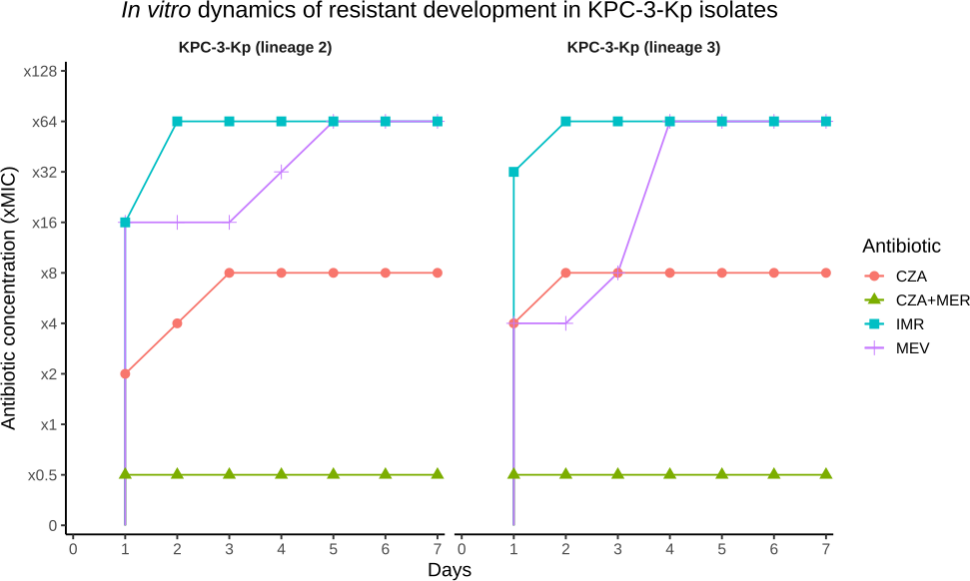
Dynamics of stepwise resistance development to CZA, ceftazidime-avibactam in combination with meropenem (CZA + MER, ratio 1:1), MEV, and IMR in broth cultures from two baseline KPC-3-ST307-*K. pneumoniae* isolates (lineages 2 and 3).

Comparative analysis of the dynamics of stepwise development of resistance to ceftazidime-avibactam, CZA + MER (ratio 1:1), meropenem-vaborbactam, and imipenem-relebactam was performed in two parental KPC-3-ST307-Kp (lineages 2 and 3). For ceftazidime-avibactam, both lineages reached 8× MIC on days 2 or 3 and remained at that level until day 7. For meropenem-vaborbactam and imipenem-relebactam, resistance developed more rapidly, reaching ≥64× MIC within the first 2–3 days and persisting until the end of the experiment. In contrast, no increase in the inhibitory concentration was observed for the CZA + MER combination over 7 days in either isolate, indicating complete suppression of resistance development under the conditions tested (Fig. 3).

### Characterization of resistant mutants

Resistant mutants selected under ceftazidime-avibactam, meropenem-vaborbactam, and imipenem-relebactam exposure during the MPC and resistance dynamics assays were subsequently analyzed by MIC testing and whole-genome sequencing to identify resistance-associated genetic alterations.

In MPC-derived mutants, mutations in plasmid *tra* genes (type F conjugative transfer system and pili modification), *xerD* (site-specific recombinase, suggesting modified integration, excision, and inversion of defined DNA segments), or *nrdR* (transcription factor, suggesting modified expression) were identified in ceftazidime-avibactam-, meropenem-vaborbactam-, and imipenem-relebactam-selected mutants, respectively. However, no relevant changes in MICs were observed (ceftazidime-avibactam: 2/4–8/4 mg/L; meropenem-vaborbactam ≤0.06/8 mg/L; and imipenem-relebactam: 0.25/4–1/4 mg/L) (Table S3).

Regarding the mutants obtained during the *in vitro* evolution experiments, genomic analysis of ceftazidime-avibactam-evolved mutants revealed that ceftazidime-avibactam resistance was primarily associated with mutations in *bla*_KPC_, which appeared from day 2 or 3 onwards in both lineages. In lineage 3, all ceftazidime-avibactam-resistant mutants carried the same *bla*_KPC_ variant (KPC-85), whereas in lineage 2, multiple distinct *bla*_KPC_ alleles (KPC-53, KPC-3[A171D], KPC-3[D178N]) emerged over time, indicating divergent evolutionary trajectories under identical selective conditions. In lineage 2, the mutants from days 6 and 7, despite growing at high ceftazidime-avibactam concentrations (64 mg/L), had lost the *bla*_KPC_-carrying plasmid, and mutations were only detected in genes related to galactose metabolism (*galE, galK,* and *galT*). Besides *bla*_KPC_, recurrent mutations were also detected in genes related to conjugative transfer (*tra* genes) or type VI secretion system (*hcpA* gene) (Table 4; Table S4). Three ceftazidime-avibactam-evolved mutants carried non-synonymous substitutions in PBP3-FtsI (ceftazidime target) and showed the largest increases in cefepime-taniborbactam MICs (Table 4; Table S4).

**TABLE 4:** Comparative phenotypic and genotypic data of the parental KPC-3-ST307-Kp and the mutants obtained on day 7 during the *in vitro* evolution experiments after exposure to CZA, MEV, and IMR^*a*^

KPC-3-Kp parental isolate	ATB pressure	Mutant ID	MIC-CZA (mg/L)	MIC-MER (mg/L)	MIC-MEV (mg/L)	MIC-IMP (mg/L)	MIC-IMR (mg/L)	MIC-FDC (mg/L)	MIC-AZA (mg/L)	MIC-FTB (mg/L)	KPC variant	Non-synonymous mutations
Lineage 2		Parental	4/4	16	≤0.06/8	8	0.25/4	1	≤0.25/4	0.5/4	KPC-3	
	CZA	L2-CZA_D7-1	0.5/4	≤0.06	≤0.06/8	≤0.25	0.5/4	0.25	≤0.25/4	0.5/4	No KPC	GalE (E157K)
		L2-CZA_D7-2	64/4	0.25	≤0.06/8	≤0.25	0.5/4	2	1/4	0.5/4	KPC-3 (D178N)	GalE (E157K)
		L2-CZA_D7-3	0.5/4	≤0.06	≤0.06/8	≤0.25	0.25/4	0.5	≤0.25/4	0.5/4	No KPC	GalE (E157K)
	MEV	L2-MEV_D7-1	2/4	>32	2/8	>32	4/4	1	1/4	8/4	KPC-3	OmpK36 (Y74X)^*b*^, AstB (A160T), Lon (A134V), tyrosin kinase (V4fs)
		L2-MEV_D7-2	2/4	>32	1/8	>32	4/4	1	1/4	4/4	KPC-3	OmpK36 (Y74X)^*b*^, AstB (A160T), Lon (A134V), Transposase IS1 X 2 (H83Y), tyrosin kinase (V4fs)
		L2-MEV_D7-3	4/4	>32	1/8	>32	4/4	0.5	≤0.25/4	4/4	KPC-3	OmpK36 (Y74X)^*b*^, AstB (A160T), Lon (A134V), tyrosin kinase (V4fs)
	IMR	L2-IMR_D7-1	64/4	>32	>16/8	>32	>8/4	1	8/4	16/4	KPC-3	OmpK36 (Y74X)^*b*^, AstB (A160T), Lon (A134V), Transposase IS1 X 2 (H83Y), LacI (V225G), PsiE (Q101X), tyrosin kinase (V4fs)
		L2-IMR_D7-2	32/4	>32	8/8	>32	>8/4	2	16/4	16/4	KPC-3	OmpK36 (Y74X)^*b*^, AstB (A160T), Lon (A134V), Transposase IS1 X 2 (H83Y), LacI (V225G), MepS (A182P), tyrosin kinase (V4fs)
		L2-IMR_D7-3	32/4	>32	>16/8	>32	>8/4	2	16/4	16/4	KPC-3	OmpK36 (Y74X)^*b*^, AstB (A160T), Lon (A134V), tyrosin kinase (V4fs)
Lineage 3		Parental	2/4	8	≤0.06/8	8	0.25/4	0.25	≤0.25/4	0.25/4	KPC-3	
	CZA	L3-CZA_D7-1	32/4	4	≤0.06/8	8	0.25/4	0.5	1/4	0.5/4	KPC-85	HcpA (L60Q)
		L3-CZA_D7-2	32/4	2	≤0.06/8	8	0.25/4	0.5	0.5/4	1/4	KPC-85	Transposase IS1 X 2 (H83Y), HcpA (L60Q)
		L3-CZA_D7-3	32/4	2	≤0.06/8	8	0.5/4	0.5	0.5/4	0.5/4	KPC-85	Transposase IS1 X 2 (H83Y), HcpA (L60Q)
	MEV	L3-MEV_D7-1	8/4	>32	4/8	>32	4/4	1	1/4	8/4	KPC-3	OmpK36 (Y74X)^*b*^, AstB (A160T), Lon (A134V), Transposase IS1 X 2 (H83Y), tyrosin kinase (V4fs)
		L3-MEV_D7-2	4/4	>32	4/8	>32	4/4	0.5	2/4	8/4	KPC-3	OmpK36 (Y74X)^*b*^, AstB (A160T), Lon (A134V), tyrosin kinase (V4fs)
		L3-MEV_D7-3	4/4	>32	4/8	>32	4/4	0.5	1/4	8/4	KPC-3	OmpK36 (Y74X)^*b*^, AstB (A160T), Lon (A134V), LldD (E114K), tyrosin kinase (V4fs)
	IMR	L3-IMR_D7-1	64/4	>32	16/8	>32	>8/4	4	16/4	32/4	KPC-3	OmpK36 (Y74X)^*b*^, AstB (A160T), Lon (A134V), tyrosin kinase (V4fs)
		L3-IMR_D7-2	64/4	>32	>16/8	>32	>8/4	4	16/4	32/4	KPC-3	OmpK36 (Y74X)^*b*^, AstB (A160T), Lon (A134V), Transposase IS1 X 2 (H83Y), tyrosin kinase (V4fs)
		L3-IMR_D7-3	64/4	>32	8/8	>32	>8/4	4	8/4	16/4	KPC-3	OmpK36 (Y74X)^*b*^, AstB (A160T), Lon (A134V), LacI (V225G), tyrosin kinase (V4fs)

^*a*^ CZA, ceftazidime-avibactam; MER, meropenem, MEV; meropenem-vaborbactam; IMP, imipenem; IMR, imipenem-relebactam; FDC, cefiderocol; AZA, aztreonam-avibactam; and FTB, cefepime-taniborbactam.

^*b*^ Resistant mutants produced an OmpK36 porin with multiple amino acid substitutions starting at position 60 (G60M), followed by a premature stop (Tyr74X).

By contrast, all meropenem-vaborbactam- and imipenem-relebactam-selected mutants retained an intact *bla*_KPC-3._ Instead, from day 2 and coinciding with MIC increases, all mutants carried a truncating mutation in the porin OmpK36 (Y74X or Y25X), consistent with complete loss of this porin. Furthermore, meropenem-vaborbactam- and imipenem-relebactam-resistant mutants were found to have mutations in *astB* (which encodes an enzyme in the arginine succinyltransferase pathway), *lon* (an ATP-dependent endopeptidase/protease), *lacI* (lactose operon repressor), or/and predicted tyrosine kinase (Table 4; Table S4).

**Fig 4:**
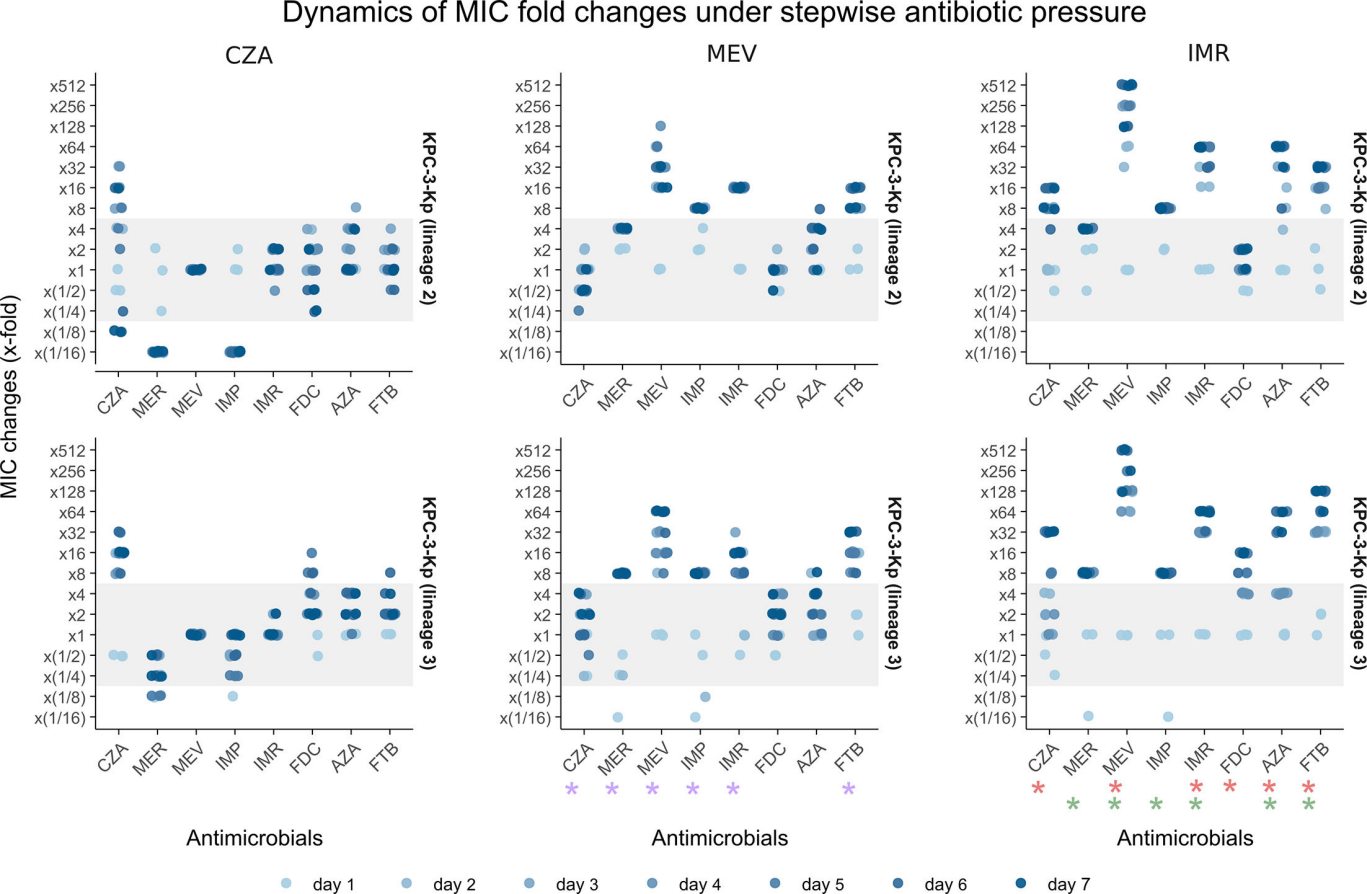
Dynamics of MIC-fold changes in mutants selected under stepwise antibiotic pressure during the *in vitro* resistance development experiment performed over seven consecutive days in two baseline KPC-3-ST307-*K. pneumoniae* strains (lineages 2 and 3) with CZA, MEV, and IMR. MICs were determined for each mutant against: CZA, MER, MEV, IMP, IMR, FDC, AZA, and FTB. The shaded gray area indicates the range corresponding to twofold changes above and below the MIC value of the corresponding parental KPC-3-ST307-*K. pneumoniae* strain. Values of ×(1/16) represent mutants showing at least a 16-fold decrease in MIC compared to the parental strain. Significant pairwise differences obtained by one-way ANOVA with Tukey’s test and Kruskal–Wallis/Wilcoxon analyses between the impact of pressure with CZA, MEV, and IMR on the log_2_-fold MIC change of each tested antimicrobial are indicated with an asterisk (purple: MEV > CZA; red: IMR > MEV; and green: IMR > CZA).

Regarding the MIC-fold changes in the obtained mutants, those selected with meropenem-vaborbactam and imipenem-relebactam were associated with significantly larger MIC increases for most novel antimicrobials compared to ceftazidime-avibactam. Additionally, imipenem-relebactam notably increased the MICs of meropenem-vaborbactam, imipenem-relebactam, aztreonam-avibactam, and cefepime-taniborbactam compared to meropenem-relebactam. Cefiderocol activity remained generally preserved in all mutants (Fig. 4). The pairwise differences in MIC changes for each antibiotic tested among the three combinations are shown in Table S5.

### Plasmid copy number and MIC changes

The relative copy number of *bla*_KPC_- and *bla*_CTX-M-15_-encoding plasmids in mutants obtained under stepwise selection was estimated (Fig. S2). In ceftazidime-avibactam-selected mutants, the relative copy number of pKPC showed a weak and non-significant correlation with changes in the ceftazidime-avibactam MIC (Spearman’s, ρ = 0.22, *P* = 0.19; linear model, β = 0.26, *P* = 0.69) (Tables S5 and S6). Note that under ceftazidime-avibactam selection, several ceftazidime-avibactam mutants lost the pCTX-M plasmid (18/42, 42.9%). In the multivariate linear model, larger reductions in pCTX-M copy number were associated with higher ceftazidime-avibactam MICs (β = −3.1, *P* = 0.03) (Table S7). Consistently, mutants that lost pCTX-M-15 displayed greater ceftazidime-avibactam MIC increases than those retaining the plasmid (Tables S3 and S4).

In meropenem-vaborbactam-selected mutants, changes in pKPC and pCTX-M-15 copy number showed a weak to moderate positive correlation with changes in meropenem-vaborbactam MIC (pKPC [Spearman’s, ρ = 0.35, *P* = 0.03]; pCTX-M-15 [Spearman’s, ρ = 0.31, *P* = 0.047]). However, in the linear model that included both pKPC and pCTX-M plasmids, neither plasmid was independently associated with meropenem-vaborbactam MIC (*P* > 0.25), and the model explained only about 10% of the variance, suggesting that plasmid copy number amplification makes only a minor contribution to meropenem-vaborbactam resistance (Tables S5 and S6). In the case of imipenem-relebactam, changes in plasmid copy number had no detectable impact on imipenem-relebactam MIC changes (Spearman’s, ρ < 0.30 and *P* > 0.07, respectively), and the linear model that included both plasmids had virtually no explanatory power (adjusted *R*² <0) (Tables S6 and S7).

MPC-derived mutants often showed marked amplification of pKPC, although no changes were detected in the CMIs compared to the parental strains (Table S2).

## DISCUSSION

In the last decades, the efficacy of antimicrobials has been compromised by not only the widespread acquisition of genes encoding resistance but also the selection of spontaneous mutations under antibiotic pressure. Although ceftazidime-avibactam, meropenem-vaborbactam, and imipenem-relebactam are among the preferred options for severe KPC-Kp infections, guidelines and reviews caution that resistance may emerge during therapy, especially under monotherapy (28). In this sense, the superiority of combination therapies based on ceftazidime-avibactam plus carbapenems over single-agent treatment for KPC-Kp infection has already been documented, in both *in vitro* and murine models of infection (13, 29, 30). Thus, the combination CZA + MER may constrain evolutionary escape, as mutations responsible for a reduced susceptibility to ceftazidime-avibactam can simultaneously increase susceptibility to meropenem, reducing the number of viable evolutionary trajectories available for resistance selection. In this work, we demonstrated that the CZA + MER combination exhibited bactericidal and synergistic activity in a clinical collection of KPC-Kp strains, including KPC-3-Kp and KPC-Kp mutants with different genetic backgrounds, even at subinhibitory concentrations. Although these conditions do not represent optimal therapeutic exposures, subinhibitory concentrations were included as a comparative *in vitro* approach to explore regrowth, resistance emergence, and synergy under partial selective pressure.

Meropenem also showed bactericidal activity at high exposures (2× MIC or 4× MIC) in all KPC-Kp clinical isolates, including those combining mutations in porin proteins or PBP2. Clinical success of meropenem has been reported in the treatment of infections by ceftazidime-avibactam-resistant isolates that remain susceptible to carbapenems (16, 31). Pariona *et al*. recently demonstrated the efficacy of meropenem in *in vitro* time-kill and *in vivo Galleria mellonella* survival models against several KPC-Kp mutants (14). However, other studies have shown that a carbapenem-resistant phenotype can be restored due to the coexistence of mixed subpopulations, the selection of mutations in OmpK36 porin, or other genomic adaptations (13, 32–34). Therefore, the use of carbapenems to treat infections by carbapenem-susceptible and ceftazidime-avibactam-resistant KPC-Kp has been recommended in combination with other non-beta-lactam antibiotics, or in high dose with extended perfusion combined with other active agents (15, 16). In this context, the CZA + MER combination, by combining synergy and collateral sensitivity, may represent a more sustainable therapeutic strategy, by preventing selection of resistant subpopulations and limiting further ceftazidime-avibactam resistance while offering practical advantages over non-β-lactam-containing combinations that carry greater toxicity or administration restrictions.

Interestingly, ceftazidime-avibactam also showed good bactericidal activity at high concentrations (2× MIC and 4× MIC) across all KPC-Kp mutants, including those containing porin alterations and mutations in PBP2 that had the highest ceftazidime-avibactam MICs (64/4 to >128/4 mg/L). Meropenem-vaborbactam was generally bactericidal, but only at higher concentrations and with slightly lower efficiency in mutants with non-functional porin proteins and higher MICs (1/8–4/8 mg/L), whereas imipenem-relebactam was bactericidal in only less than half of the isolates, including the two porin-deficient strains (imipenem-relebactam MIC: 2/4 mg/L).

In strains where bactericidal activity of meropenem-vaborbactam and imipenem-relebactam was not observed, bacterial regrowth occurred after 7 h, especially at high antibiotic concentrations. These findings should be interpreted considering β-lactams’ pharmacodynamics: they exhibit time-dependent killing, and efficacy correlates with the fraction of the dosing interval that free drug concentrations exceed the MIC (%fT >MIC). Maintaining adequate redosing and/or prolonged high-dose infusions has been shown to be the most effective approach to ensuring optimal PK/PD targets with both carbapenems and novel BL/BLI combinations, thereby minimizing resistance selection and the risk of clinical failure (35, 36). In our collection, KPC-Kp isolates exhibited low MICs to meropenem-vaborbactam and imipenem-relebactam, especially those strains with intact porins. Because our static model lacked re-dosing, drug concentrations were not maintained and quickly dropped below the MIC, enabling regrowth. In contrast, higher concentrations of ceftazidime-avibactam and CZA + MER better maintain bactericidal activity. However, it should be noted that for ceftazidime-avibactam, the 2 × MIC and 4 × MIC exposures required for the highest MIC mutants are unlikely to be sustained *in vivo* with approved dosing regimens. Nevertheless, for meropenem-vaborbactam and imipenem-relebactam, activity at 2 × MIC may be clinically plausible in isolates displaying low MIC values under optimized infusion regimens, whereas sustained 4 × MIC exposures should be interpreted cautiously, particularly in the absence of redosing or prolonged infusion.

The CZA + MER combination showed superior *in vitro* efficacy during *in vitro* evolution experiments and showed a mutation rate <10^−10^, preventing the selection and evolution of resistant subpopulations in both KPC-ST307-Kp lineages. By contrast, a rapid stepwise selection of resistant populations was observed with ceftazidime-avibactam, meropenem-vaborbactam, and imipenem-relebactam. Notably, resistance to meropenem-vaborbactam and imipenem-relebactam developed more rapidly and at lower concentrations than to ceftazidime-avibactam.

The clinical success of both ceftazidime-avibactam and meropenem-vaborbactam is similar in patients with recurrent infection by KPC-Kp, but ceftazidime-avibactam is more frequently used as combination therapy to avoid the *in vivo* resistant development (9). In fact, previous studies have reported a lower risk of emergence of resistance during meropenem-vaborbactam therapy than with ceftazidime-avibactam (9, 22, 37, 38). Sun *et al*. showed that meropenem-vaborbactam-resistant mutants can be prevented by using exposures achieved with the optimal dosing of the combination (22). Interestingly, in our baseline ST307-KPC-3-Kp strains, the MPC of meropenem-vaborbactam (0.5/8 mg/L) was lower than the MPC of ceftazidime-avibactam (8/4 mg/L). However, meropenem-vaborbactam displayed higher mutation frequencies (10⁻⁵−10⁻⁶) than ceftazidime-avibactam (≤10⁻⁸). Note that these values for meropenem-vaborbactam were also higher than those reported by Sun *et al*. (≤10⁻⁸) (22). These discrepancies could reflect the higher frequency of porin mutations under exposure to carbapenems (but not ceftazidime). Furthermore, the clinical origin of our isolates or intrinsic characteristics of the ST307 clone, such as a higher basal mutation rate or a clone-specific resistome/permeability, could also be contributing factors.

Recent studies also support imipenem-relebactam as a good therapeutic option for KPC-Kp, including ceftazidime-avibactam-resistant isolates (8, 39, 40). However, its effectiveness has been questioned due to the selection of resistant mutants from KPC-Kp in both ceftazidime-avibactam susceptible and resistant strains under antibiotic pressure (23, 24). *In vivo* evolution of resistance to imipenem-relebactam has also been reported in KPC-Kp isolates following prolonged ceftazidime-avibactam and meropenem-vaborbactam exposure (25). In our ST307-KPC-Kp strains, the MPC of imipenem-relebactam (4/4 mg/L) was comparable to those of ceftazidime-avibactam (8/4 mg/L), and mutation frequencies for imipenem-relebactam were slightly higher (~10⁻⁷) than that of ceftazidime-avibactam (≤10⁻⁸). A similar mutation frequency for ceftazidime-avibactam resistance (~10^−9^) had previously been described in KPC-Kp by Livermore *et al*. (~10^−9^) (41). Remarkably, the imipenem-relebactam mutation rate in our KPC-3-ST307-Kp strains was higher than that previously reported by Blanco-Martín *et al*. in KPC-ST512-Kp and KPC-ST258-Kp mutants (~10^−8^–10⁻^10^) (40). The large window of selection of imipenem-relebactam (MIC: 0.25/4 mg/L; MPC: 4/4 mg/L) could explain the higher frequency of emergence and selection of resistant mutants compared to ceftazidime-avibactam, particularly when exposure is continuous. Our results were consistent with Gato *et al*., who showed that imipenem-relebactam-resistant mutants are selected at lower concentrations than with ceftazidime-avibactam, although with a higher biological cost that may limit their persistence (24).

WGS analysis of mutants obtained during the evolution experiments highlights a different adaptive potential of KPC-ST307-Kp strains when exposed to different combinations. Ceftazidime-avibactam resistance emerged predominantly through mutations in *bla*_KPC_, consistent with previous clinical and experimental evidence (11, 12). The rapid emergence and diversification of *bla*_KPC_ variants underscore the strong selective pressure exerted by ceftazidime-avibactam and the ability of KPC-3 to explore multiple mutational pathways. Note that ceftazidime has previously been identified as the key diversification driver of other carbapenemases, as VIM-type beta-lactamases (42). Furthermore, mutations in *tra* genes or in the type VI secretion system further suggested additional adaptations affecting plasmid transfer/maintenance and cell envelope-associated functions. In contrast with previous studies, increased *bla*_KPC_ gene expression or a higher copy number of the *bla*_KPC_ plasmid did not lead to a higher level of ceftazidime-avibactam resistance in our mutants (25, 43, 44). In fact, late-stage ceftazidime-avibactam-resistant mutants from one lineage lost the *bla*_KPC_ plasmid and had mutations on the *gal* operon, suggesting that under prolonged ceftazidime-avibactam pressure, resistance could be maintained likely via additional chromosomal adaptations, as previously reported in OXA-48-producing *K. pneumoniae* (45). Loss of the *bla*_CTX-M-15_ plasmid was also observed in mutants with the highest ceftazidime-avibactam MICs, supporting a possible fitness cost associated with plasmid maintenance under antibiotic pressure.

By contrast, meropenem-vaborbactam and imipenem-relebactam did not lead to KPC diversification, but instead rapidly selected truncating mutations in OmpK36. Previous studies have shown OmpK35/OmpK36 porin alterations together with increased *bla*_KPC_ copy number as the main drivers of resistance to meropenem-vaborbactam and imipenem-relebactam in *K. pneumoniae* (21–25). In our mutants, multiple copies of *bla*_KPC_ were not detected, and *bla*_KPC_ plasmid copy number had a minor or non-detectable contribution to resistance. Additional mutations in *astB*, *lon*, *lacI*, and a predicted tyrosine kinase were found, suggesting that metabolic and regulatory adaptations may further contribute to bacterial fitness under antibiotic pressure, although this requires functional validation.

Cross-resistance patterns also differed according to the selection combination. Previous studies have reported collateral resistance to cefiderocol and other BL/BLI combinations in ceftazidime-avibactam-resistant mutants with *bla*_KPC_ variants, frequently carrying additional mutations affecting porin proteins or iron transporters (17, 18, 26, 46). In our study, ceftazidime-avibactam selection resulted only in increases in ceftazidime-avibactam MICs, and in some cases, collateral sensitivity to meropenem and imipenem. In contrast, meropenem-vaborbactam and imipenem-relebactam selected broader cross-resistance, including aztreonam-avibactam and cefepime-taniborbactam, both targeting MBL-producing bacteria. Our findings are supported by previous studies that have reported that pressure with imipenem-relebactam causes increased MICs to ceftazidime-avibactam, meropenem-vaborbactam, and aztreonam-avibactam (23, 24). Notably, cross-resistance was significantly greater with imipenem-relebactam than with meropenem-vaborbactam, despite similar genetic alterations. This likely reflects the intrinsic pharmacodynamic characteristics of imipenem-relebactam, which achieves greater affinity and more sustained engagement with its PBP targets compared to meropenem-vaborbactam, thereby exerting stronger selective pressure and extending collateral effects to other porin-dependent β-lactams such as aztreonam-avibactam and cefepime-taniborbactam. In contrast, the activity of cefiderocol remained largely preserved, consistent with its primary uptake via siderophore-mediated iron transport systems rather than classical porins.

This study has some limitations. First, it was exclusively performed *in vitro*, and the results may not fully reproduce the complexity of *in vivo* infection settings, including host factors and dynamic antibiotic exposures. Second, only a limited number of strains were analyzed. Third, all isolates belonged to the ST307 high-risk clone, which may restrict the generalizability of our findings to other *K. pneumoniae* lineages. Despite these limitations, the study provides relevant mechanistic insights into resistance emergence, mutant prevention, and collateral resistance under antibiotic pressure in a clinically relevant KPC-producing background.

In conclusion, our results demonstrate that ceftazidime-avibactam, meropenem-vaborbactam, and imipenem-relebactam generate distinct selective landscapes in KPC-producing *K. pneumoniae* ST307, leading to different resistance trajectories and collateral resistance patterns. Whereas ceftazidime-avibactam primarily selected *bla*_KPC_ diversification with limited cross-resistance, meropenem-vaborbactam and particularly imipenem-relebactam promoted rapid permeability-based adaptations that extend resistance to multiple porin-dependent β-lactams, including agents such as aztreonam-avibactam or cefepime-taniborbactam designed for MBL-producing pathogens. Importantly, ceftazidime-avibactam plus meropenem was the only regimen that combined consistent synergistic bactericidal activity with complete prevention of the selection of resistant mutants under the conditions tested. Although these findings require confirmation *in vivo*, they support the concept that combination therapy may offer an important clinical advantage over single-agent regimens by reducing the likelihood of resistance emergence during treatment, particularly in infections with high bacterial load. Furthermore, our findings underscore that the sustainability of novel BL/BLI therapies when designing treatment strategies for KPC-Kp infections should be evaluated not only in terms of antimicrobial activity but also according to their evolutionary and collateral consequences.

## MATERIALS AND METHODS

### Bacterial isolates and study design

A total of eight ST307-*K. pneumoniae* clinical strains, one representative isolate carrying each different mutated KPC variant detected in our hospital, were selected for this study: KPC-31-ST307-Kp, KPC-46-ST307-Kp, KPC-53-ST307-Kp, KPC-61-ST307-Kp, KPC-62-ST307-Kp, KPC-66-ST307-Kp, KPC-92-ST307-Kp, and KPC-150-ST307-Kp. All isolates exhibited resistance to ceftazidime-avibactam (range: 32/4–128/4 mg/L) and were recovered from clinical or colonization samples obtained from patients admitted to our institution between 2018 and 2020. Additionally, three KPC-3-ST307-Kp clinical isolates initially susceptible to ceftazidime-avibactam (range: 2/4–4/4 mg/L) and obtained from three patients from whom ceftazidime-avibactam-resistant mutants (KPC-46-ST307-Kp, KPC-66-ST307-Kp, and KPC-92-ST307-Kp) emerged during ceftazidime-avibactam treatment were also included in the study (12). These baseline strains were used for comparative time-kill kinetics, synergy studies, and to conduct *in vitro* evolution experiments aimed at selecting resistant mutants. All KPC-3-ST307-Kp and mutated KPC-ST307-Kp strains were previously well characterized by both microbiological and molecular methods (Tables S1 and S2) (12, 18, 26, 27). TOP10**-***E. coli* transformed with plasmids carrying each one of the *bla*_KPC_ variants analyzed in this study were successfully obtained as previously described (26). MICs in all obtained transformants were also measured in triplicate using broth microdilution by SENSITITRE EUMDROXF (ThermoFisher, US). This study was approved by the Ramón y Cajal University Hospital Ethics Committee (reference 293/19).

### Antimicrobial susceptibility testing

MIC determination for ceftazidime-avibactam, MER, ceftazidime-avibactam in combination with MER (ratio 1:1), imipenem-relebactam, and meropenem-vaborbactam was performed using broth microdilution in accordance with ISO 20776-2:2021 (47). For novel β-lactam/β-lactamase inhibitor combinations, inhibitors were tested at fixed concentrations: avibactam and relebactam at 4 mg/L, and vaborbactam at 8 mg/L. Susceptibility testing for additional agents, including imipenem, cefepime, piperacillin-tazobactam, aztreonam, cefotaxime, aztreonam-avibactam, cefepime-taniborbactam, and cefiderocol, was also performed using broth microdilution (SENSITITRE EUMDROXF panel, ThermoFisher Scientific, Inc., Cleveland, OH). All results were interpreted according to EUCAST-2025 criteria (https://www.eucast.org/clinical_breakpoints).

### Killing kinetics studies

Killing curves were performed with all KPC-ST307-Kp mutants and one baseline KPC-3-ST307-Kp strain using the drop test (48). We evaluated the activity of ceftazidime-avibactam, meropenem, CZA + MER, imipenem-relebactam, and meropenem-vaborbactam. The tested antibiotic concentrations were as follows: 0.5× MIC value, MIC value, 2× MIC, and 4× MIC. β-lactamase inhibitors were used at fixed concentrations: avibactam and relebactam at 4 mg/L, and vaborbactam at 8 mg/L. Each tube was inoculated with a 10^6^ CFU/mL suspension to obtain a final inoculum of approximately 10^5^ CFU/mL. The *in vitro* activity was assessed at 0 h, 1 h, 3 h, 5 h, 7 h, and 24 h. At each timepoint, 10 µL from six serial 1/10 dilutions and the original medium from the tubes were plated as a spot onto Mueller-Hinton agar (MHA) plates. After incubating at 37°C for 24 h and 48 h, the colony growth in the spots was counted, and the results were plotted as log_10_ CFU/mL at each hour. A reduction of the original inoculum by ≥3 log_10_ CFU/mL was considered bactericidal. For the combination of CZA + MER, synergy was defined as a ≥ 2 log_10_ CFU/mL decrease at 24 h for the antimicrobial combination compared with the most active single agent.

### Synergy testing

MIC gradient strip (Etest, BioMerieux, France) synergy test was performed to study the synergistic effect of ceftazidime-avibactam in combination with meropenem. All KPC-ST307-Kp mutants and one baseline KPC-3-ST307-Kp strain were used. MICs were determined on MHA plates using gradient strips crossed perpendicularly at the intersection between MICs of antibiotics. Plates were incubated at 37°C for 24 h. Results were expressed as the fractional inhibitory concentration (FIC) index [ΣFIC = (MIC-A in combination/MIC-A alone) + (MIC-B in combination/MIC-B alone)]. A ΣFIC ≤0.5 was considered as synergistic, 0.5< ΣFIC ≤4 as indifferent, and ΣFIC >4 as antagonistic (48).

### Single-step mutant selection and MPC assays

The MPC was determined for ceftazidime-avibactam, CZA + MER (ratio 1:1), imipenem-relebactam, and meropenem-vaborbactam using two KPC-3-ST307-Kp baseline strains. Briefly, initial inocula of 10^9–10^ CFU/mL from an overnight culture were plated onto MHA plates supplemented with the antibiotic at increasing concentrations (MIC value, 2× MIC, 4× MIC, 8× MIC, and 16× MIC). For meropenem, meropenem-vaborbactam, and imipenem-relebactam, assays were additionally performed with an initial inoculum adjusted to 10⁶ CFU/mL to enable reliable colony quantification. The mutation frequency was calculated as the fraction of resistant mutants (median number of mutants/median total cell number). The MPC was defined as the lowest concentration of antibiotic that inhibited all visible growth (zero colonies) after 48 h of incubation at 37°C. Up to four colonies per strain and antibiotic growing below the MPC were further analyzed by standard broth microdilution, PCR, and Sanger sequencing to confirm them as potential KPC-Kp mutants. The potential emergence of collateral resistance to cefiderocol, aztreonam-avibactam, and/or cefepime-taniborbactam was also assessed.

### Dynamics of *in vitro* resistance development

A comparative analysis of the dynamics of the gradual development of resistance to ceftazidime-avibactam, CZA + MER, imipenem-relebactam, and meropenem-vaborbactam was performed in two KPC-3-ST307-Kp parental strains following the protocol previously described by Cabot *et al*. (49). Tubes with 10 mL of Mueller-Hinton broth were inoculated with 10^6^ CFU/mL of an exponentially growing KPC-3-Kp clinical strain. After 24 h of incubation at 37°C and at 180 rpm, the culture with the highest antibiotic concentration showing visible growth was reinoculated at a 1:1,000 dilution ratio in fresh tubes containing incremental concentrations up to 64× MIC. This process was repeated until growth was extinguished or visible growth was observed at MIC ≥ 64 mg/L. To detect resistant mutants, 100 μL was taken from the last tube showing visible growth, as well as from all subsequent tubes with higher antibiotic concentrations and no visible growth, and plated onto antibiotic-free MHA. After 24-h incubation at 37°C, up to three colonies per strain, antibiotic, and day were selected for further characterization from the plate corresponding to the highest antibiotic concentration from which colony growth was recovered. MICs were re-determined by microdilution and PCR, and Sanger sequencing was performed to confirm them as potential KPC-Kp mutants. Collateral sensitivity to carbapenems was defined as inhibition of growth of ceftazidime-avibactam-resistant KPC-Kp mutants by meropenem or imipenem at MIC values at least two dilution steps (fourfold) lower than those of the ceftazidime-avibactam-susceptible KPC-3-Kp parental strain (50). Kaplan–Meier curves were plotted to represent the survival or extinction of bacterial populations. The development of collateral resistance to cefiderocol, aztreonam-avibactam, and/or cefepime-taniborbactam was also investigated.

### Whole-genome sequencing and bioinformatic analysis

Selected mutants obtained during the resistance dynamics (three mutants per strain, antibiotic, and day) and MPC assays (four mutants per strain, antibiotic, and day) from baseline KPC-3-Kp strains were analyzed and compared to their corresponding parental strains by WGS. Genomic DNA extraction was performed using the QIAamp DNA Mini Kit QIAgen (Qiagen, GmbH, Hilden, Germany). Short-read sequencing was performed using the Illumina NovaSeq 6000 or MiSeq (UCA-GT, University Hospital Ramón y Cajal, Madrid, Spain) platforms with 2 × 150 bp paired-end reads. Quality control and sequence processing were carried out as previously described (18). KPC-3-ST307-Kp parental isolates were also analyzed using Oxford-Nanopore (MinION) technology. From these strains, complete hybrid assemblies were obtained from the combination of short and long reads using Unicycler, Flye, and Pilon tools (18). Parental strains were annotated by Prokka and subsequently used as reference genomes for variant calling analysis. SNPs and small insertions and deletions (InDels) between the mutant isolates and their corresponding annotated reference genomes were extracted using Snippy. Estimated plasmid copy number (coverage ratio, plasmid/chromosome) was calculated (51).

All complete sequences were deposited at DDBJ/ENA/GenBank under project numbers PRJNA637023, PRJNA741867, PRJNA747261, PRJNA985214, PRJNA1205681) (Table S1). GenBank accession numbers of the novel *bla*_KPC-3_ genes are PX663403 (KPC-3-D178N) and PX663404 (KPC-3-A171D).

### Statistical analysis

For each tested antibiotic (ceftazidime-avibactam, meropenem-vaborbactam, imipenem-relebactam, imipenem, meropenem, cefiderocol, aztreonam-avibactam, and cefepime-taniborbactam), log_2_-fold MIC changes were compared across selecting antibiotics (ceftazidime-avibactam, meropenem-vaborbactam, imipenem-relebactam) using one-way ANOVA followed by Tukey’s test. Kruskal–Wallis tests with pairwise Wilcoxon rank-sum comparisons (Benjamini–Hochberg correction) were also performed as a non-parametric sensitivity analysis.

Relative copy numbers of the plasmids carrying *bla*_KPC_ and *bla*_CTX-M-15_ were estimated as the ratio of plasmid to chromosomal read coverage and expressed as log_2_ fold-change *versus* the parental isolate (51). Associations between log_₂_-fold MIC and log_₂_-fold plasmid copy number changes were first assessed using Spearman’s rank correlation. For each selecting antibiotic (ceftazidime-avibactam, meropenem-vaborbactam, imipenem-relebactam), a multivariable linear model was fitted including pKPC and pCTX-M copy number as predictors. Models included all mutants obtained with both lineages during the evolution experiments additionally adjusted for selecting the drug as a categorical covariate. All analyses were performed in R.

## Data Availability

All complete genome sequences were deposited at DDBJ/ENA/GenBank under project numbers PRJNA637023, PRJNA741867, PRJNA747261, PRJNA985214, and PRJNA1205681.

## SUPPLEMENTAL MATERIAL

Supplemental material aac.00488-26-s0001.docx Tables S1 and S2, S5 to S7; Fig. S1 and S2.

Supplemental tables aac.00488-26-s0002.xlsx Tables S3 and S4.

ASM does not own the copyrights to Supplemental Material that may be linked to, or accessed through, an article. The authors have granted ASM a non-exclusive, world-wide license to publish the Supplemental Material files. Please contact the corresponding author directly for reuse.
