# Supplementary material for: Addressing therapeutic options for KPC-3-producing ST307-Klebsiella pneumoniae: insights from in vitro evolution and mutant prevention strategies
Source: Antimicrob Agents Chemother. 2026 Jul 1;70(8):e00488-26. doi: 10.1128/aac.00488-26 (PMC13436382; doi:10.1128/aac.00488-26)
Supplement: Supplementary file 1 [file aac.00488-26-s0001.docx]

**SUPPLEMENTARY MATERIAL**

**Addressing therapeutic options for KPC-3-producing ST307-*Klebsiella pneumoniae*: insights from *in vitro* evolution and mutant prevention strategies**

**Marta Hernández-García^1,2*^, Blanca Pérez-Viso^1,2^, Marta Nieto-Torres^1^, María García Castillo^1,2^, Malkoa Michelena^1^, Luis Martínez-Martínez^2,3^, Fernando Baquero^1^, Patricia Ruiz-Garbajosa^1,2^*, Rafael Cantón^1,2^**

^1^Servicio de Microbiología, Hospital Ramón y Cajal and Instituto Ramón y Cajal de Investigación Sanitaria (IRYCIS), Madrid, Spain.

^2^CIBER de Enfermedades Infecciosas (CIBERINFEC), Instituto Salud Carlos III (ISCIII), Madrid, Spain.

^3^Unidad de Microbiología, Hospital Universitario Reina Sofia. Departamento de Química Agrícola, Edafología y Microbiología, Universidad de Córdoba e Instituto Maimónides de Investigación Biomédica de Córdoba (IMIBIC), Cordoba, Spain.

^4^CIBER de Epidemiología y Salud Pública (CIBERESP), Instituto Salud Caelos III (ISCIII), Madrid, Spain.

**Running title**: Therapeutic options for KPC-*K. pneumoniae* infections

**Key-words**: KPC-ST307-*K. pneumoniae*; ceftazidime-avibactam; meropenem-vaborbactam; imipenem-relebactam; collateral sensitivity to carbapenems

***Corresponding authors**: Patricia Ruiz-Garbajosa ([pruizg@salud.madrid.org](mailto:pruizg@salud.madrid.org)) and Marta Hernández-García ([martahernandez1986@gmail.com](mailto:martahernandez1986@gmail.com)). Hospital Universitario Ramón y Cajal. Carretera de Colmenar Km 9,1. 28034-Madrid. Spain

| **Table S1**. Epidemiological and molecular data of KPC-ST307-Kp clinical isolates selected for the study. | | | | | | | |
| --- | --- | --- | --- | --- | --- | --- | --- |
| **Isolate** | **Year of isolation** | **Source** | **Phenotype**  **profile** | **KPC mutation** | **Other *bla* genes** | **Chromosomal mutations*** | **Accession number** |
| KPC-31-ST307-Kp | 2019 | Rectal | ESBL-*like* | D179Y | *bla*_CTX-M-15_, *bla*_TEM-1B_, *bla*_SHV-28_, *bla*_OXA-1_ | *mdrA/pbp2* (A523T) | [JAHWYW000000000](https://www.ncbi.nlm.nih.gov/nuccore/JAHWYW000000000) |
| KPC-46-ST307-Kp | 2020 | Rectal | ESBL-*like* | L168P | *bla*_CTX-M-15_, *bla*_TEM-1B_, *bla*_SHV-28_, *bla*_OXA-1_, *bla*_OXA-9_ |  | [JAHUYZ000000000](https://www.ncbi.nlm.nih.gov/nuccore/JAHUYZ000000000) |
| KPC-53-ST307-Kp | 2020 | Catheter | ESBL-*like* | L167E168dup | *bla*_CTX-M-15_, *bla*_TEM-1B_, *bla*_SHV-28_, *bla*_OXA-1_, *bla*_OXA-9_ |  | [JBNSZW000000000](https://www.ncbi.nlm.nih.gov/nuccore/JBNSZW000000000) |
| KPC-61-ST307-Kp | 2018 | Surgical wound | ESBL-*like* | S170P | *bla*_CTX-M-15_, *bla*_TEM-1B_, *bla*_SHV-28_, *bla*_OXA-1_, *bla*_OXA-9_ |  | [JABUIO000000000](https://www.ncbi.nlm.nih.gov/nuccore/JABUIO000000000) |
| KPC-62-ST307-Kp | 2020 | Urine | Carbapenemase-*like* | L168Q | *bla*_CTX-M-15_, *bla*_TEM-1_, *bla*_SHV-28_, *bla*_OXA-1_, *bla*_OXA-9_ | *ompA* (S138F), *ompR/envZ* (Y52C, Q103L) | [JAHWYR000000000](https://www.ncbi.nlm.nih.gov/nuccore/JAUEGS000000000) |
| KPC-66-ST307-Kp | 2020 | Rectal | ESBL-*like* | E167_L168del | *bla*_CTX-M-15_, *bla*_TEM-1B_, *bla*_SHV-28_, *bla*_OXA-1_, *bla*_OXA-9_ |  | [JAHUYX000000000](https://www.ncbi.nlm.nih.gov/nuccore/JAHUYX000000000) |
| KPC-92-ST307-Kp | 2020 | Blood | ESBL-*like* | E167_N169delinsD | *bla*_CTX-M-15_, *bla*_TEM-1B_, *bla*_SHV-28_, *bla*_OXA-1_, *bla*_OXA-9_ |  | [JAHUYV000000000](https://www.ncbi.nlm.nih.gov/nuccore/JAHUYV000000000) |
| KPC-150-ST307-Kp | 2020 | Organic fluid | Carbapenemase-*like* | L166_N169delinsH | *bla*_CTX-M-15_, *bla*_TEM-1B_, *bla*_SHV-28_, *bla*_OXA-1_, *bla*_OXA-9_ | *truncated OmpK36, OmpK35* (Q350*) | [JAUEHB000000000](https://www.ncbi.nlm.nih.gov/nuccore/JAUEHB000000000) |
| KPC-3-ST307-Kp (lineage 1) | 2020 | Bronchial aspirate | Carbapenemase-*like* | - | *bla*_CTX-M-15_, *bla*_TEM-1B_, *bla*_SHV-28_, *bla*_OXA-1_, *bla*_OXA-9_ |  | [JAHUZA000000000](https://www.ncbi.nlm.nih.gov/nuccore/JAHUZA000000000) |
| KPC-3-ST307-Kp (lineage 2) | 2020 | Rectal | Carbapenemase-*like* | - | *bla*_CTX-M-15_, *bla*_TEM-1_, *bla*_SHV-28_, *bla*_OXA-1_, *bla*_OXA-9_ |  | [JAHUYY000000000](https://www.ncbi.nlm.nih.gov/nuccore/JAHUYY000000000) |
| KPC-3-ST307-Kp (lineage 3) | 2020 | Rectal | Carbapenemase-*like* | - | *bla*_CTX-M-15_, *bla*_TEM-1B_, *bla*_SHV-28_, *bla*_OXA-1_, *bla*_OXA-9_ |  | [JAHUYW000000000](https://www.ncbi.nlm.nih.gov/nuccore/JAHUYW000000000) |
| *Mutations in genes previously associated with resistance to novel beta-lactamase/beta-lactamase inhibitor combinations and/or cefiderocol were analyzed in KPC-Kp mutants and compared to the corresponding genes in previous KPC-3-ST307-Kp isolates (lineage 1, 2 and 3): membrane permeability (*ompK35*, *ompK36*), siderophores (*fiu*), TonB-dependent energy transduction complex (*tonB, exbB, exbD*), PBP (*ftsI-pbp3, mdrA-pbp2*), enterobactin or ferrochrome receptors (*fhuA, fepA*), efflux transporter (*sugE, chrA, yicM-nepI*), other (*baeS/R, envZ-ompR, ompA*) (Hernández-García *et al*. J Clin Microbiol. 2022 Mar 16;60(3):e0224521. doi: 10.1128/jcm.02245-21.). | | | | | | | |

| **Table S2**. Antimicrobial susceptibility results of KPC-producing TOP10*-E. coli* transformants obtained by cloning experiments and heat shock transformation (11, 26). | | | | | | | | | | | | | | | |
| --- | --- | --- | --- | --- | --- | --- | --- | --- | --- | --- | --- | --- | --- | --- | --- |
| **Transformants^a^** | **MIC values (mg/L)^b^** | | | | | | | | | | | | | | |
|  | **PT4** | **FEP** | **AZT** | **CT** | **CZA** | **FDC CMI** | **IMI** | **IMR** | **MERO** | **MEV** | **TGC** | **ERV** | **COL** | **AMI** | **TOB** |
| **TOP10-*E.coli*-pKPC-3** | >32/4 | >16 | >32 | >8/4 | 1/4 | 0.5 | >8 | 0.5/4 | >16 | ≤0.06/8 | ≤0.5 | 0.06 | ≤0.5 | ≤2 | ≤0.5 |
| **TOP10-*E.coli*-pKPC-31** | ≤4/4 | ≤1 | ≤1 | >8/4 | >16/4 | 0.5 | ≤1 | 0.25/4 | ≤0.12 | ≤0.06/8 | ≤0.5 | 0.06 | ≤0.5 | ≤2 | ≤0.5 |
| **TOP10-*E.coli*-pKPC-46** | 8/4 | 8 | 16 | 8/4 | >16/4 | 1 | ≤1 | 0.25/4 | ≤0.12 | ≤0.06/8 | ≤0.5 | 0.06 | ≤0.5 | ≤2 | ≤0.5 |
| **TOP10-*E.coli*-pKPC-61** | 8/4 | 4 | 8 | >8/4 | >16/4 | 1 | ≤1 | 0.25/4 | 0.25 | ≤0.06/8 | ≤0.5 | 0.06 | ≤0.5 | ≤2 | ≤0.5 |
| **TOP10-*E.coli*-pKPC-62** | 8/4 | 4 | 8 | 8/4 | 16/4 | 1 | ≤1 | 0.25/4 | ≤0.12 | ≤0.06/8 | ≤0.5 | 0.06 | ≤0.5 | ≤2 | ≤0.5 |
| **TOP10-*E.coli*-pKPC-92** | 8/4 | 4 | 2 | 8/4 | 16/4 | 1 | ≤1 | 0.25/4 | ≤0.12 | ≤0.06/8 | ≤0.5 | 0.06 | ≤0.5 | ≤2 | ≤0.5 |
| **TOP10-*E.coli*-pKPC-150** | 8/4 | 16 | 16 | >8/4 | >16/4 | 2 | ≤1 | 0.25/4 | ≤0.12 | ≤0.06/8 | ≤0.5 | 0.06 | ≤0.5 | ≤2 | ≤0.5 |
| PT4: piperacillin-tazobactam, FEP: cefepime, CT: ceftolozane/tazobactam, CZA: ceftazidime/avibactam, FDC: cefiderocol, IMI: imipenem, IMR: imipenem/relebactam, MER: meropenem, MEV: meropenem/vaborbactam, AZT: aztreonam, TGC: tigecycline, ERV: eravacycline, COL: colistin, , AMK: amikacin, TOB: tobramycin.  ^a^KPC-53 and KPC-66-producing TOP10-*E. coli* transformants were not obtained  ^b^MIC values obtained using the EUMDROXFTM panel. | | | | | | | | | | | | | | | |

**Figure S1**. Results of time–kill assays of KPC-46-ST307-Kp, KPC-53-ST307-Kp, KPC-61-ST307-Kp, KPC-66-ST307-Kp and KPC-92-ST307-Kp strains exposed to meropenem (MER), ceftazidime-avibactam (CZA), ceftazidime-avibactam in combination with meropenem (CZA+MER; ratio 1:1), meropenem-vaborbactam (MEV) and imipenem-relebactam (IMR) at increasing concentrations (0.5xMIC, 1xMIC, 2xMIC and 4xMIC). Growth control represents the mean value (log_10_ CFU/mL) of all strains in the absence of antibiotics. For all assays, the initial inoculum was ∼10^5^ CFU/mL.

| **Table S5.** Pairwise comparisons of log₂-fold MIC changes between selection conditions: ceftazidime-avibactam (CZA), meropenem-vaborbactam (MEV) and imipenem-relebactam (IMR). | | | | |
| --- | --- | --- | --- | --- |
| **Tested antibiotic** | **Comparison (group2 - group1)** | **Δlog₂(MIC) Tukey** | **p-Tukey (adj)** | **p-Wilcoxon (BH)** |
| **CZA** | **MEV - CZA** | **-2.31** | **7.0×10⁻⁷** | **5.3×10⁻⁶** |
|  | IMR - CZA | -0.05 | 0.993 | 0.76 |
|  | **IMR - MEV** | **2.26** | **1.2×10⁻⁶** | **2.9×10⁻⁶** |
| **MEV** | **MEV - CZA** | **4.10** | **<1×10⁻¹²** | **8.2×10⁻¹⁴** |
|  | **IMR - CZA** | **6.50** | **<1×10⁻¹²** | **8.2×10⁻¹⁴** |
|  | **IMR - MEV** | **2.40** | **6.0×10⁻⁷** | **5.3×10⁻⁸** |
| **IMR** | **MEV - CZA** | **3.02** | **<1×10⁻¹²** | **2.4×10⁻¹¹** |
|  | **IMR - CZA** | **4.48** | **<1×10⁻¹²** | **2.4×10⁻¹¹** |
|  | **IMR - MEV** | **1.45** | **4.48×10⁻⁵** | **1.3×10⁻⁸** |
| **IMP** | **MEV - CZA** | **4.69** | **<1×10⁻¹²** | **1.6×10⁻¹³** |
|  | **IMR - CZA** | **4.83** | **<1×10⁻¹²** | **2.5×10⁻¹⁴** |
|  | IMR - MEV | 0.14 | 0.93 | 0.75 |
| **MER** | **MEV - CZA** | **5.74** | **<1×10⁻¹²** | **3.2×10⁻¹³** |
|  | **IMR - CZA** | **5.88** | **<1×10⁻¹²** | **9.6×10⁻¹⁴** |
|  | IMR - MEV | 0.14 | 0.95 | 0.76 |
| **FDC** | MEV - CZA | -0.40 | 0.33 | 0.11 |
|  | IMR - CZA | 0.60 | 0.088 | 0.17 |
|  | **IMR - MEV** | **1.00** | **0.00146** | **0.0083** |
| **AZA** | MEV - CZA | 0.05 | 0.99 | 0.88 |
|  | **IMR - CZA** | **2.76** | **<1×10⁻¹²** | **2.8×10⁻⁷** |
|  | **IMR - MEV** | **2.71** | **<1×10⁻¹²** | **2.8×10⁻⁷** |
| **FTB** | **MEV - CZA** | **2.67** | **<1×10⁻¹²** | **3.4×10⁻¹¹** |
|  | **IMR - CZA** | **3.79** | **<1×10⁻¹²** | **3.4×10⁻¹¹** |
|  | **IMR - MEV** | **1.12** | **0.00167** | **2.7×10⁻⁵** |
| Pairwise differences were supported by a one-way ANOVA with Tukey's test and confirmed by the Kruskal-Wallis/Wilcoxon tests.  Outcome: log₂-fold MIC change vs parental isolate. Differences are expressed as mean Δlog₂(MIC) (group2 − group1). Significant comparisons (p < 0.05 in both tests) are shown in bold. | | | | |

**Figure S2**. Relative pKPC and pCTX-M-15 plasmid copy number during stepwise selection with ceftazidime-avibactam (CZA), meropenem-vaborbactam (MEV) and imipenem-relebactam (IMR) in two different lineages. Values are shown as fold-change versus the parental isolate (log₂-scale in analyses; raw fold-change shown here).

| **Table S6**. Spearman correlation between Δlog₂(MIC) and Δlog₂(copy number). | | | |
| --- | --- | --- | --- |
| **Selecting antibiotic** | **Plasmid** | **Spearman ρ** | **p-value** |
| **CZA** | pKPC | 0.2226 | 0.1919 |
|  | pCTX-M-15 | 0.0145 | 0.9462 |
| **MEV** | pKPC | 0.3473 | **0.0281** |
|  | pCTX-M-15 | 0.3117 | **0.04725** |
| **IMR** | pKPC | -0.2959 | 0.07533 |
|  | pCTX-M-15 | -0.1652 | 0.2959 |
| Only evolution-line mutants (days 1–7) were included in the statistical analyses. | | | |

| **Table S7**. Linear models Δlog₂(MIC) ~ Δlog₂(copy number pKPC + pCTX-M-15). | | | | | |
| --- | --- | --- | --- | --- | --- |
| 2A. Model coefficients | | | | | |
| **Selecting antibiotic** | **Predictor** | **Estimate** | **Std. Error** | **t value** | **p-value** |
| **CZA** | Intercept | 0.1290 | 0.8868 | 0.145 | 0.8863 |
|  | l2_pKPC | 0.2587 | 0.6351 | 0.407 | 0.6895 |
|  | l2_pCTXM | -3.0869 | 13.176 | -2.343 | **0.0333** |
| **MEV** | Intercept | 40.896 | 0.4108 | 9.955 | 7.01e-12 |
|  | l2_pKPC | 0.2989 | 0.6746 | 0.443 | 0.660 |
|  | l2_pCTXM | 16.793 | 15.117 | 1.111 | 0.274 |
| **IMR** | Intercept | 48.168 | 0.3603 | 13.369 | 4.27e-15 |
|  | l2_pKPC | -0.2361 | 0.4990 | -0.473 | 0.639 |
|  | l2_pCTXM | 0.4855 | 14.374 | 0.338 | 0.738 |
| 2B. Global adjustment of each model | | | | | |
| **Selecting antibiotic** | **n (efective)** | **R²** | **Adjusted R²** | **F (df = 2, n−3)** | **p-value (model)** |
| **CZA** | 18 | 0.3238 | 0.2336 | 3.591 | 0.05317 |
| **MEV** | 39 | 0.1057 | 0.05598 | 2.127 | 0.134 |
| **IMR** | 37 | 0.00656 | -0.05187 | 0.1123 | 0.8941 |
| Only evolution-line mutants (days 1–7) were included in the statistical analyses. p-values > 0.05 are shown in bold. | | | | | |
